# Heterotaxy syndrome with agenesis of dorsal pancreas and diabetes mellitus: case report and review of the literature

**DOI:** 10.20945/2359-3997000000142

**Published:** 2019-05-25

**Authors:** Cínthia Minatel Riguetto, Samantha Pelichek, Arnaldo Moura

**Affiliations:** 1 Universidade Estadual de Campinas Universidade de Campinas Faculdade de Ciências Médicas Divisão de Endocrinologia Campinas SP Brasil Divisão de Endocrinologia, Faculdade de Ciências Médicas, Universidade de Campinas, Campinas, SP, Brasil

## Abstract

Heterotaxy syndrome (HS) is a rare congenital condition with multifactorial heritance, characterized by an abnormal arrangement of thoraco-abdominal organs and vessels. Patients present with multiple cardiac, gastrointestinal, hepatosplenic, pancreatic, renal, neurological and skeletal disorders without any pathognomonic alteration. Despite the described increased risk of diabetes mellitus (DM) in patients with altered pancreatic anatomy, just one case was reported in Korea regarding the association of HS and DM in a 13-year-old girl. Our report refers to a 40-year-old female Brazilian patient with a history of DM and HS with polysplenia and agenesis of dorsal pancreas without cardiac abnormalities. She presented a worsening glycemic control associated with weight gain and signs of insulin resistance. After a proper clinical management of insulin and oral medications, our patient developed an improvement in glycemic control. Although it is a rare disease, HS with polysplenia and pancreatic disorders can be associated with an increased risk of DM. This case highlights the importance of investigating DM in patients with HS, especially those with pancreatic anatomical disorders, for proper clinical management of this rare condition.

## INTRODUCTION

Heterotaxy syndrome (HS) is a rare congenital condition with multifactorial inheritance characterized by an abnormal arrangement of thoraco-abdominal organs and vessels. Patients present with multiple cardiac, gastrointestinal, hepatosplenic, pancreatic, renal, neurological and skeletal disorders without any pathognomonic alteration (1–3). The first case was described in 1929 and the disease has an incidence rate of 1 in 15,000 live births. HS has two main classifications: HS with polysplenia, which presents with thoraco-abdominal abnormalities and multiple spleens; and HS with asplenia (4,5). Patients with polysplenia eventually have short pancreas or agenesis of the dorsal pancreas, a feature related to an increased risk of pancreatitis and diabetes mellitus – DM (6,7). Despite the described increased risk of DM, we found just one case report associated with DM in a 13-year-old girl with HS and pancreatic disorder (8). We report a case of a 40-year-old female patient with HS and polysplenia in association with DM due to agenesis of dorsal pancreas.

## CASE REPORT

A 40-year-old non-Caucasian female Brazilian patient presented to the Endocrinology Division of the University Hospital at Campinas in June 2017, complaining of fatigue, polyphagia, polyuria, polydipsia and weight loss (5 kg) for 30 days.

The patient had been diagnosed with DM, hypertension, dyslipidemia and HS four years earlier and was currently using metformin 2 g/day, gliclazide 90 mg/day, enalapril 20 mg/day, hydrochlorothiazide 25 mg/day and simvastatin 20 mg/day. She worked as a secretary for about 60 hours a week, did not do exercises and had a high carbohydrate intake diet, especially with industrialized products. She did not have any family history of DM or other relevant diseases. Information retrieved from her medical history showed good glycemic control and body weight until July 2016, when the patient presented a glycosylated hemoglobin (HbA1c) of 6.6%, fasting plasma glucose (FPG) of 143 mg/dL, weight of 66 kg and body mass index (BMI) of 22.83 kg/m^2^. However, in March 2017, those parameters increased, and she presented an HbA1c of 8.3%, FPG of 174 mg/dL, weight of 76 kg and BMI of 26.29 kg/m^2^.

**Figure 1:**
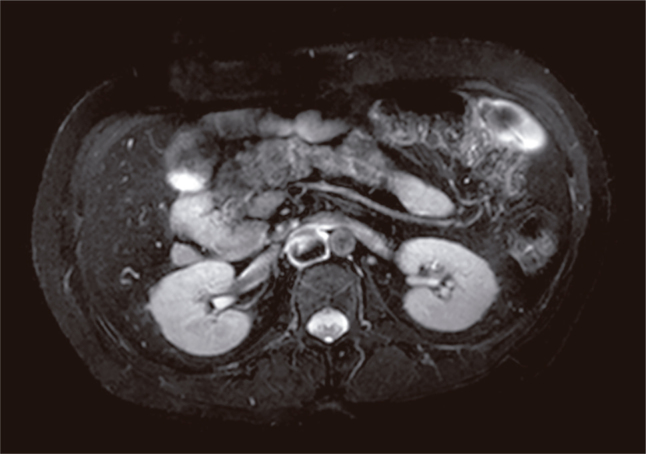
Abdominal MRI and cholangiopancreatography MRI showing agenesis of dorsal pancreas with increased dimensions of the pancreas head.

In further investigation from medical records, an abdominal magnetic resonance imaging (MRI) and a cholangiopancreatography MRI from March 2016 showed agenesis of dorsal pancreas with increased dimensions of the pancreas head (Figure 1). The liver was enlarged and presented an ectopic location, situated in a central position in the abdomen. Also, there were signs of non-homogenous steatosis. Intestinal malrotation was also seen, with the stomach and angle of Treitz located to the right, with a predominance of small intestines in the right hemiabdomen. The spleen was dislocated from the usual position. In the right hypochondrium, there were multiple nodular images with lobulated contours, similar to splenic parenchyma. No renal, gallbladder or aortic disorders were found. An echocardiogram did not show any alterations in cardiac position or function.

On clinical examination, the patient presented with a weight of 71 kg, height of 1.70 m, BMI of 24.56 kg/m^2^, blood pressure of 120/80 mmHg, 89 heart beats/minute and waist circumference of 98 cm. Acanthosis nigricans was noted in the cervical, axillary and inguinal regions. No alteration was found during the pulmonary and cardiologic exam. Upon abdominal examination, a parenchymal consistency mass was palpated in the mesogastric region.

Blood tests showed an HbA1c of 10.2%, FPG 142 mg/dL, C-peptide 0.91 ng/mL (normal range 0.8-4.2 ng/mL), microalbuminuruia 60.9 mg/L, aspartate aminotransferase (AST) 37 U/L (normal range < 35 U/L), alanine aminotransferase (ALT) 65 U/L (normal range < 35 U/L), alkaline phosphatase (ALP) 137 U/L (normal range 30-120 U/L) and gamma-glutamyl transferase (GGT) 730 U/L (normal range 9-64 U/L). The tittles of autoantibodies (islet cell antibody, anti-glutamine acid decarboxylase antibody and anti-insulin antibody) were negative.

Oral medications were discontinued, and NPH and regular insulin were started and progressively increased. The patient achieved a total dose of 120 IU per day to obtain good glycemic control associated with nutritional education and less industrialized carbohydrate intake. After 30 days of well-controlled blood glucose levels, metformin 1.5 grams/day was reintroduced and sitagliptin 100 mg/day added to the regimen, since the patient had evident signs of insulin resistance, such as acanthosis nigricans and increased abdominal circumference. Two months after the reintroduction of oral medications, the total insulin dose had been reduced to 30 IU per day.

Three months after the first appointment, the patient reported an improvement in asthenia, tiredness, and quality of life, mainly because she was sleeping better since the polyuria stopped. On clinical examination, she presented with a weight of 77 kg, waist circumference of 102 cm, BMI 26.64 kg/m^2^ and her HbA1c decreased to 6.8%. Other laboratorial parameters were also improved: FPG 145 mg/dL, C-peptide 2.45 ng/mL, AST 25 U/L, ALT 41 U/L, ALP 131 U/L and GGT 429 U/L. Evolution of clinical and laboratorial parameters are described in Table 1. The patient maintains regular clinical follow-ups with an endocrinologist, nutritionist, and gastroenterologist to control all the abnormalities associated with the syndrome.

**Table 1:** Clinical and laboratory parameters before the first evaluation and in subsequent visits

Clinical and Laboratory Parameters	July 2016	March 2017	June 2017	October 2017
Weight (kg)	66	76	71	77
Body Mass Index (kg/m^2^)	22.83	26.29	24.56	26.64
Waist circumference (cm)			94	102
Fast plasma glucose (RV 65-99 mg/dL)	143	174	142	145
Glycosylated hemoglobin (%)	6.6%	8.3%	10.2%	6.8%
C-peptide (RV 0.8-4.2 ng/mL)			0.91	2.45
Antiglutamine acid decarboxylase antibody (RV < 10 UI/mL)			Negative	
Islet cell antibody (RV Non-reagent)			Negative	
Anti-insulin antibody (RV < 8.2%)			Negative	
Total cholesterol (RV < 200 mg/dL)	176	203	282	163
LDL (RV < 130 mg/dL)	94	109	158	80
HDL (RV > 50 mg/dL)	60	63	60	54
Triglycerides (RV < 150 mg/dL)	109	159	323	143
Aspartate aminotransferase (RV < 35 U/L)	42	45	37	25
Alanine aminotransferase (RV < 35 U/L)	65	72	65	41
Alkaline phosphatase (RV 30-120 U/L)	132	143	137	131
Gamma-glutamyl transferase (9-64 U/L)	699	701	730	429

The significant challenges, in this case, are mainly related to the rarity of HS and the difficulty of establishing whether it could be a case of DM type 2 in a patient with reduced pancreas or if diabetes was only secondary to pancreas agenesis.

## DISCUSSION

HS is an anomaly due to a defect of lateralization during the early embryonic process, with an approximated incidence rate of 1 in 15,000 live births. It is more prevalent in men, at a ratio of 2:1 (4,9,10). This inability of lateralization is most often sporadic, but genetic inheritance has been proposed, such as autosomal dominant, recessive and X-linked recessive inheritance. Recently, human genetics studies have also revealed some genes that are responsible for left-right laterality and HS, such as ZIC3, NODAL, and LEFTY2 (11,12).

Heterotaxy with polysplenia, as in our case, is the most common type of HS and has an increased frequency in women. Commonly, it is characterized by multiple spleens associated with a pattern of abnormalities in several other systems (13,14). However, these patients may also have a single-lobed spleen or even a normal spleen. Affected patients have a low prevalence of congenital heart diseases and less severe defects than patients with HS and asplenia, but when cardiac lesions are associated with this syndrome, the 1-year mortality rate can rise up to 50% (15–17).

The exact prevalence of pancreatic disorders related to HS with polysplenia is not known, but in a series of eight cases, Gayer and cols. (18) reported that four patients had a short pancreas. Dorsal pancreatic agenesis is usually an asymptomatic condition and may be found incidentally, during the investigation of other diseases. However, patients may complain of abdominal pain and present hyperglycemia. Hyperglycemia is seen in approximately 50% of cases, owing to loss of islet cells that are mainly found in the tail and body of the pancreas (19,20).

Jung and cols. (8) described the first case of DM due to agenesis of dorsal pancreas in a 13-year-old girl with HS and several cardiac and extracardiac disorders. Our patient, differently from the first case report, has HS with polysplenia and agenesis of the dorsal pancreas, but we did not find any cardiac abnormality. She developed DM at 36 years of age and had a transitory worsening in glycemic control four years after the diagnosis, in association with clinical signs of insulin resistance (acanthosis nigricans and increased waist circumference) and weight gain.

Initially, the patient presented a low C-peptide level and required high doses of insulin, but after an improvement in glycemic control, we were able to restart oral medications (metformin and sitagliptin) and decrease insulin doses. The evaluation of clinical characteristics, especially the signs of insulin resistance and the level of peptide C, was important to guide the decision to restart oral medications in an attempt to improve glycemic sensitivity in a patient with altered pancreatic anatomy.

It is quite difficult to affirm the etiology for the worsening in glycemic control observed in this patient. It could be due to a natural evolution of the disease or, more likely, to the progressive weight gain and consequent insulin resistance being responsible for the worsening in glycemic control in a patient with a diminished pancreatic reserve due to the loss of islet cells that predominate in the body and tail of pancreas (21).

In conclusion, although HS with polysplenia and pancreatic disorder is a rare anomaly, it can be associated with an increased risk of pancreatitis and DM. While a case of HS with dorsal pancreas agenesis and DM was already described, we present a different type of HS coexisting with polysplenia, agenesis of dorsal pancreas and DM in a 40-year-old woman. This case highlights the importance of investigating DM in patients with HS, especially those with pancreatic anatomical disorders, for proper clinical management of this rare condition.
